# Comparison between multiple-trait and random regression models for genetic evaluation of weight traits in Australian meat sheep

**DOI:** 10.1093/jas/skae038

**Published:** 2024-02-09

**Authors:** Uddhav Paneru, Nasir Moghaddar, Julius van der Werf

**Affiliations:** School of Environment and Rural Science, University of New England, NSW 2351, Armidale, Australia; School of Environment and Rural Science, University of New England, NSW 2351, Armidale, Australia; School of Environment and Rural Science, University of New England, NSW 2351, Armidale, Australia

**Keywords:** accuracy, forward prediction, multiple-trait model, random regression, slope

## Abstract

Random regression (RR) models are recommended as an alternative to multiple-trait (MT) models for better capturing the variance–covariance structure over a trajectory and hence more accurate genetic evaluation of traits that are repeatedly measured and genetically change gradually over time. However, a limited number of studies have been done to empirically compare RR over a MT model to determine how much extra benefit could be achieved from one method over another. We compared the prediction accuracy of RR and MT models for growth traits of Australian meat sheep measured from 60 to 525 d, using 102,579 weight records from 24,872 animals. Variance components and estimated breeding values (EBVs) estimated at specific ages were compared and validated with forward prediction. The accuracy of EBVs obtained from the MT model was 0.58, 0.51, 0.54, and 0.56 for weaning, postweaning, yearling, and hogget weight stages, respectively. RR model produced accuracy estimates of 0.56, 0.51, 0.54, and 0.54 for equivalent weight stages. Regression of adjusted phenotype on EBVs was very similar between the MT and the RR models (*P* > 0.05). Although the RR model did not significantly increase the accuracy of predicting future progeny performance, there are other benefits of the model such as no limit to the number of records per animal, estimation of EBVs for early and late growth, no need for age correction. Therefore, RR can be considered a more flexible method for the genetic evaluation of Australian sheep for early and late growth, and no need for age correction.

## Teaser

For genetic evaluation, a random regression model can accommodate multiple measures of body weight on an animal and is flexible in fitting these exactly according to their age at measurement, following a smooth trajectory of variance and covariance. However, this feature was shown to have limited added value in increasing the accuracy of EBVs compared to a multiple-trait model that assumes measurement at fixed ages.

## Introduction

Weight traits are among the lamb industry’s most studied traits because they are easily measured and have high economic importance in the commercial sheep production system. In the Australian national genetic evaluation for meat sheep (LAMBPLAN), weight measurements are evaluated as discrete traits. This includes birth, weaning (Wwt), postweaning (Pwt), yearling (Ywt), hogget (Hwt), and adult weights, each with a defined age range (Table 1). Multiple-trait (MT) genetic analysis is carried out to calculate the estimated breeding value (EBV) of these traits and these EBVs are used to make selection decisions. The age range for each trait is broad, and hence a pre-correction of weight records for age is needed for each trait to account for age differences within each trait (Brown et al., 2000, 2016). MT genetic evaluation for weight allows to estimate EBVs of all animals even if animals do not have a record at a certain weight stage, as information from pedigree and genetic correlations between traits can be used. Further, the MT model gives a more accurate genetic evaluation by using information from different ages to estimate EBVs for each age stage. However, demarcating weight into different traits based on discrete age classes may not be optimal and could affect the accuracy of estimating genetic parameters and EBVs.

**Table 1.** Summary of the data

Description	Summary	Description	Summary
Number of animals	24,827	Animals with 5 records	3,251
Number of records	102,579	Animal with 6 records	10
Animals in the pedigree	58,081	Wwt-age (days)	60 to 165
Sires with progeny records	1,600	Records for Wwt	24,710
Dams with progeny records	14,443	Pwt-age (days)	105 to 345
^1^Number of CG	3,255	Records for Pwt	24,339
Number of flocks	79	Ywt-age (days)	285 to 435
Mean weight in Kg (±SD)	51.87 ± 18.20	Records for Ywt	21,861
Animals in the training	21,168	Hwt-age (days)	375 to 525
^2^Progeny’s used in FP	3,561	Records for Hwt	9,275
Animals with four records	21,566		

^1^CG = Contemporary group; ^2^FP = Animals used for forward prediction and were born after 2016

Weight can be repeatedly measured at different ages, but the underlying genetic mechanisms may gradually change with age (Albuquerque and Meyer, 2001; Schaeffer, 2004). The function that describes weight changes over time is of interest to animal breeders since it may help us understand, explain, and manipulate how that characteristic changes genetically over time and therefore predict the effect of selection, i.e., how it changes the growth curve. Kirkpatrick and Heckman (1989) defined a function to describe multiple measures of a phenotype of a trajectory, for which they used the term infinite-dimensional characters and explained the benefits of using an infinite-dimensional model over finite conventional methods. A model that deals with repeated measurements over a trajectory can be the random regression model (RR).

The RR model is advocated as a better model than the MT model, e.g., for genetic evaluation of weight traits in sheep (Lewis and Brotherstone, 2002; Fischer et al., 2004; Saghi et al., 2018). Further, Meyer (2004) observed higher accuracy of 0.023, 0.031, and 0.034, equivalent to average percentage increase in accuracy of 4.3, 5.6, and 5.9 for 200, 400, and 600 d weight in beef cattle by switching from an MT to an RR analysis, using both simulated and field data. Meyer (2004) advocated that the RR model achieved higher accuracies than the MT model due to better modeling of variance and the genetic parameters. The correlation between EBVs obtained from the MT and RR models was 0.81 and 0.87 for weight at weaning and postweaning in Australian sheep, indicating a significant difference in EBV ranking (Fischer et al., 2004).

Most of the research work on the RR model focuses on the estimation of genetic parameters and comparing equivalent genetic parameters with those from the MT model. Few studies have empirically compared the prediction accuracies of RR and MT models based on large field data sets in sheep. When it comes to selecting a method for genetic evaluation, the criterion of the predictive ability of a model is much more relevant than just comparing the genetic parameters estimated via these models.

The main aim of this study was to evaluate the accuracy of EBVs of a RR model for weight traits compared to a MT model. For that purpose, we estimated genetic parameters of weight traits from the weaning to hogget stage using the RR model and compared these with equivalent genetic parameters obtained from the MT model. The correlation of EBVs obtained by those two methods was assessed to determine how re-ranking EBVs occurs between the two methods and the predictive ability of the two methods was compared empirically through forward prediction.

## Materials and Methods

### Data

**Figure 1.**
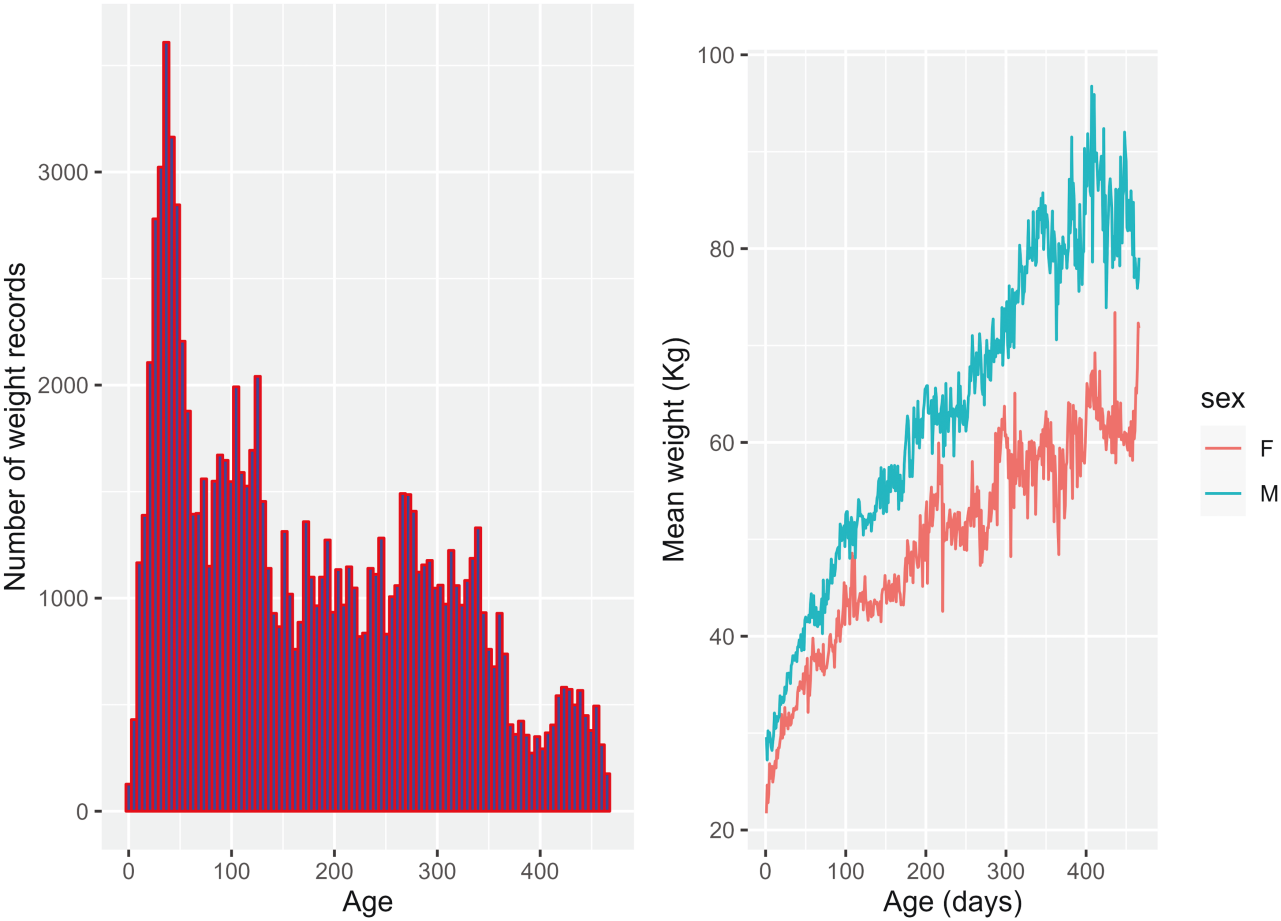
Histogram of a number of weight records across age on the left, and mean weight of animals across age for male and female on the right.

Data were extracted from the LAMBPLAN database (Brown et al., 2007), comprising weight records for Australian and New Zealand sheep. Data from terminal sire breeds were extracted, which constitutes White Suffolk, Poll Dorset, Texel, and Suffolk including animals born between 2009 and 2019. Body weight records taken outside the age range of 60 to 525 d of age were removed because of fewer records across these ages. Animals with at least four weight records were selected for the analysis because the benefit from the RR model can be achieved in animals with repeated records. Analysis with both RR and MT models had equal numbers of animals, but each animal had 1 to 4 records in the MT model because only a single record for each weight stage was considered for the analysis, excluding multiple weight records on a single stage. Data were filtered following the standard OVIS filters (Brown et al., 2000) as follows. Body weight records outside the range of 15 to 120 kg were removed. Records outside the age of the dam range of 0.8 to 12 yr were also removed. Lambs with birth type and rear type of 5 were merged with the group of lambs with birth type 4 and rear type 4, respectively, because of few animals with a birth/rear type of 5. All animals considered for the analysis had records on age of measurements, birth type, rear type, age of the dam, and have their sire and dam recorded, i.e., their full pedigree was known. The back pedigree of the animals up to the fourth generation was used during the analysis. A concise summary of the final data is presented in Table 1 and Figure 1.

### MT model

The mixed-effects model used to estimate the fixed and random effects solutions is as follows:


$$\begin{array}{l} y=Xb+Z_{1\;}a+Z_{2}m+Z_{3}mp+Z_{1}Qd+e \end{array}$$ (1)

where **y** is the vector of observations for the trait of interest, **b** is the vector of fixed effects, **a** is the vector of EBVs, **m** is the vector of maternal breeding values, **mp** is the vector of maternal permanent environmental effects, and **d** is a vector of breed effects. ***X*** is the incidence matrix relating **b** to **y** and ***Z***_**1**_, ***Z***_**2**_, and ***Z***_**3**_ and ***Z***_**1**_ are incidence matrices relating **a**, **m**, **mp**, **d** to **y** and **e** is the vector of random residuals. ***Q*** is a matrix containing breed-group proportion coefficients for each animal in the pedigree. Matrix ***Q*** was created based on a pedigree with base animal groups by breed and year of birth. There were 73 genetic groups at the time of analysis. Fixed effects fitted were contemporary group (CG), birth type, rear type, age of the measurements, and age of the dam as a linear and quadratic component for weight traits. Four weight traits were analyzed with the MT model: Wwt, Pwt, Ywt, and Hwt, with the description of traits summarized in Table 1.

### RR model

#### Fixed effects

Weight was modeled as fixed quadratic regression on orthogonal polynomials of age in days. A CG effect, defined as a combination of breed, flock, management group, sex, and day of measurement, was fitted with Legendre polynomial of order 1 (i.e., intercept and slope). Other fixed effects were birth type and rear type, fitted with a Legendre polynomial of order one, and dam age with only intercept. Fixed effects models were optimized based on the partial *F* test (Gilmour et al., 2009). The partial *F* test can be defined as follows:


$$\begin{array}{l} F={\left(\frac{Solution\;of\;sum\;of\;coef\;f\;icients}{Sum\;of\;standard\;errors}\right)}^{2} \end{array}$$

If *F*_value_ > *P*_tabulated_, fixed effect is significant, else fixed effect is not significant.

The same fixed effects were fitted for all the models making restricted maximum likelihood and information criteria for the RR model of a different order of fit for random effects directly comparable. Furthermore, the same fixed effects were fitted in both MT and RR models to compare them directly, but a difference was that fixed effects in RR were regression on age, whereas in the MT model, it is not.

#### Random effects fitted for RR

Legendre polynomials of age at recording (in days) effects on all random effects were fitted as independent variables. Model building started with the simplest model, with a first-order polynomial fitted for all random effects. Higher order of polynomials was included in the subsequent model, with different orders of polynomials tested for each random effect. Polynomials fit of up to five were considered to determine the most parsimonious model describing the data.

#### Model of analysis

Let *y*_*ij*_ denotes the *j*-th records for *i*-th animal taken at time *t*_*ij*_. Then RR model on Legendre polynomials of age of recording can be defined as follows:


$$\begin{array}{llll} y_{ij}= & \;F_{ij}+\underset{m=0}{\overset{k_{P-1}}{\sum }}\;\alpha_{im}\Phi_{m}(t_{ij})+\underset{m=0}{\overset{k_{Q-1}}{\sum }}\;\gamma_{im}\Phi_{m}(t_{ij})\; & +\underset{m=0}{\overset{k_{R-1}}{\sum }}\;\delta_{im}\Phi_{m}(t_{ij})+\underset{m=0}{\overset{k_{S-1}}{\sum }}\;\rho_{im}\Phi_{m}(t_{ij})\; & +\underset{m=0}{\overset{k_{T-1}}{\sum }}\;\eta_{im}\Phi_{m}(t_{ij})+\;\;\varepsilon \;ij\;\;\;\;\;\;\; \end{array}$$

where *t*_*ij*_ indicates the standardized age at recording (−1 < *t* < 1) for *y*_*ij*_. *F*_*ij*_ denotes the fixed effects relating to *y*_*ij*_, including the fixed quadratic regression on Legendre polynomials of age. Φ_*m*_ (*t*_*ij*_) is a corresponding *m*-th order Legendre Polynomial for each random effect. Vectors **α**_*im*_, **γ**_*im*_, **δ**_*im*_, **ρ**_*im*_, and **η**_*im*_ are *m*-th order RR coefficients for direct additive genetic effect, animal permanent environmental effect, maternal genetic, maternal permanent environmental effect, and breed effects, respectively; and *k*_*P*-1_, *k*_*Q*-1_, *k*_*R*-1_, *k*_*S*-1_, and *k*_*T*-1_ are corresponding order of fit of the polynomial for each random effect and *ε*_*ij*_ is a random residual effect. Residuals are expected to vary over time and modeled using heterogeneous residual variance with 10 classes based on 47 d intervals, from the measurement’s 60 to 525 d. All analysis was carried out using ASReml 4.2 (Gilmour and Thompson, 2021).

The model in matrix notation is as follows:


$$\begin{array}{l} y=Xb+Z_{4}\alpha +Z_{5}\gamma +W_{3}\delta +W_{4}\rho +S_{2}\eta +e\; \end{array}$$

The structure of the expected values and covariance matrix is as follows:


$$\begin{array}{l} E\left[\begin{array}{l} \begin{array}{l} y\;\alpha \;\gamma \;\delta \;\rho \;\eta \;e\; \end{array}\; \end{array}\right]=\left[\begin{array}{l} \begin{array}{l} X\beta \;0\;0\;0\;0\;0\;0\; \end{array}\; \end{array}\right];\;V\left[\begin{array}{l} \begin{array}{l} \alpha \;\gamma \;\delta \;\rho \;\eta \;e\; \end{array}\; \end{array}\right]=\left|\begin{array}{lllllllllllllllllllllllllllllll} K_{P}⊗A & 0 & 0 & 0 & 0 & 0\;0 & K_{Q}⊗I & 0 & 0 & 0 & 0\;0 & 0 & K_{R}⊗A & 0 & 0 & 0\;0 & 0 & 0 & K_{S}⊗I & 0 & 0\;0 & 0 & 0 & 0 & K_{T}⊗AQ & 0\;0 & 0 & 0 & 0 & 0 & R\; \end{array}\right| \end{array}$$

where ***K***_***p***_, ***K***_***Q***_, ***K***_***R***_, ***K***_***s***_ and ***K***_***T***_ are variance and covariance matrices between respective RR coefficients, **A** is a relationship matrix, **I** is an identity matrix of order equal to the number of levels for each effect, ***Q*** is a matrix containing a breed proportion coefficient for each animal in the pedigree, ⊗ is a Kronecker product, and ***R*** is a diagonal matrix with residual variance according to the age interval in which the observation was made.

#### Model comparison

Model selection for the random effects was based on Akaike’s information criterion (AIC) to select the best-fit model (Akaike, 1973). This can be defined as follows:


$$\begin{array}{lll} AIC= & -2\log(maximum\;likelihood)\; & +2(number\;of\;parameters) \end{array}$$

Similarly, the other most popular information criterion used was Bayesian information criteria (BIC), which put a more stringent penalty on extra parameters and accounted for model uncertainty. The BIC is defined as follows:


$$\begin{array}{lll} BIC= & -2\log(maximum\;likelihood)\; & -log(n)\times (number\;of\;model\;parameters) \end{array}$$

where *n* is equal to the total number of records used in the analysis. Many authors have used AIC and BIC as model selection criteria (Meyer, 2001, 2004; Huisman et al., 2002; Fischer et al., 2004). Further, the likelihood ratio test was executed by multiplying the difference in log likelihood between the full model and reduced model by two and contrasted with the chi-square value at the α level of 5%.

#### Estimation of genetic parameters and EBVs

Variance for random effects at certain ages can be estimated by pre- and post-multiplying the covariance matrix ***K*** with a matrix of Legendre polynomial Φ, evaluated at a certain set of ages. The genetic variance–covariance matrix for breeding values for such a set of ages can be calculated as follows:


$$\begin{array}{l} G=\;\Phi K\Phi^{\prime } \end{array}$$

EBVs were calculated by multiplying the matrix of the Legendre polynomial with regression coefficients for the animal (Mrode and Thompson, 2005). EBV for the *m*-th animal can be calculated with the following formula.


$$\begin{array}{l} {\hat{u}}_{m}=\Phi k_{m}\; \end{array}$$

where ${\hat{u}}_{m}$ the EBV for *m*-th animal, Φ is a matrix of Legendre polynomial and *k*_*m*_ is the regression coefficient for each animal. Finally, EBVs is a summation of random genetic effect within-group added to genetic group solutions. Point estimates of EBVs were estimated at 100, 225, 360, and 450 d to allow EBVs from the RR to be directly comparable with EBVs estimated from the MT model at Wwt, Pwt, Ywt, and Hwt, respectively.

#### Empirical validation of EBVs accuracy and bias

Forward prediction was done to test the predictive ability of the MT and RR model to observe how well the model can empirically predict future animal performance. The full data were divided into two sets: 1) animal born from 2009 to 2016 was used as a training set and 2) animal born from 2017 to 2019 was used as a validation set. EBVs of all animals were estimated using the training set. EBVs of the animals in the validation set were estimated based on family information, without their performance records. Phenotypes of all the animals in the validation set were corrected for fixed effects from the solutions obtained from the mixed model using all data. Correlation was estimated between the EBVs of validation animals and adjusted phenotypes. Finally, accuracy is calculated according to the following formula.


$$\begin{array}{l} \frac{cor(EBV,y*\;)}{\sqrt{h^{2}}} \end{array}$$

where y* is the adjusted phenotype and $\sqrt{h^{2}\;}\;$ is the square root of the trait heritability. The slope was estimated as regressing the adjusted phenotype of validation animals on its EBVs. Slopes lower than one indicate an overdispersion of EBVs; there is less variation in the animals in the validation than is explained by the range of breeding values, while a slope greater than one suggests under dispersion of EBVs; there is more variation in animals in the validation than is explained by the range of breeding values.

Accuracy was also estimated with prediction error variance (PEV) to determine whether this method can correctly infer actual predictive ability. Accuracy estimate from PEV can be calculated as follows:


$$\begin{array}{l} r=\sqrt{\frac{1-PEV}{V_{a}}} \end{array}$$

where *r* indicates the model-based accuracy of EBVs and *V*_a_ is additive genetic variance.

## Results

### Model selection

Various models that were compared for log likelihood, corresponding information criteria and log-likelihood ratio (LRT) tests are presented in Table 2. The model building initially started with the simplest model, and a higher level of polynomials is fitted in the subsequent model. It was observed that a model, which includes a cubic Legendre polynomial for additive genetic and animal permanent environmental effects, a quadratic polynomial for maternal genetic and maternal permanent environmental effects, and a linear polynomial for genetic group effect was the optimal model, assessed based on higher log likelihood, lower AIC and BIC values. This model requires the estimation of 45 (co) variance components.

**Table 2.** Log likelihood and corresponding information criteria

Model^a^	Parameters estimated	Log likelihood	AIC	BIC	LRT relative to next model
22222	24	−171,053	342,154	342,381	
33133	35	−170,110	340,290	340,621	943 (*P* < 0.001)
33232	34	−170,110	340,288	340,610	0 (*P* > 0.05)
44331	45	−169,624	339,335	339,752	486 (*P* < 0.001)

^a^Order of fit for additive genetic effect, animal permanent environmental effect, maternal genetic effect, maternal permanent environmental effect, and genetic groups effect.

### Coefficient matrix (K)

Covariance matrices estimated for the various random effects and the pertaining eigenvalues for the selected model are presented in Table 3. Although we fitted a higher-order polynomial for the random effects, 83.92% of the variation was accounted for by one dimension. Out of all random effects fitted, animal permanent environmental effects, genetic group effects, and additive genetic effects account for most of the variation with the sum of their respective eigenvalues being 36.3, 20.48, and 17.45, respectively.

**Table 3.** Coefficient matrix and respective eigenvalues

0	1	2	3	Eigenvalues
Direct additive genetics coefficients (***K***_***P***_)				
13.79	0.75	0.11	0.217	15.36
4.45	2.53	0.34	0.06	1.21
0.34	0.44	0.69	0.18	0.64
0.55	−0.07	0.10	0.47	0.24
Animal permanent environmental coefficients (***K***_***Q***_)				
28.56	0.43	−0.05	0.23	29.76
5.35	5.24	0.22	−0.30	4.42
−0.39	0.69	1.84	0.29	1.82
1.04	−0.56	0.3	0.66	0.30
Maternal genetic (***K***_***R***_)				
3.36	−0.15	−0.33		3.37
0.08	0.09	−0.39		0.13
−0.18	−0.04	0.09		0.05
Maternal permanent environmental coefficients (***K***_***R***_)				
2.65	0.03	−0.14		2.65
0.32	0.47	0.25		0.48
−0.06	0.04	0.06		0.06
Genetic group coefficients (***K***_***T***_)				
19.63	1			20.48
4.43	0.92			0

Estimates of covariance (lower triangle) and correlations (upper triangle between RR coefficients: 0, intercept; 1, linear; 2, quadratic; 3, cubic) together with the eigenvalues (λ) of the covariance matrices, obtained by fitting Legendre polynomials with a model 44331.

### Variance parameters estimated from the RR model

**Figure 2.**
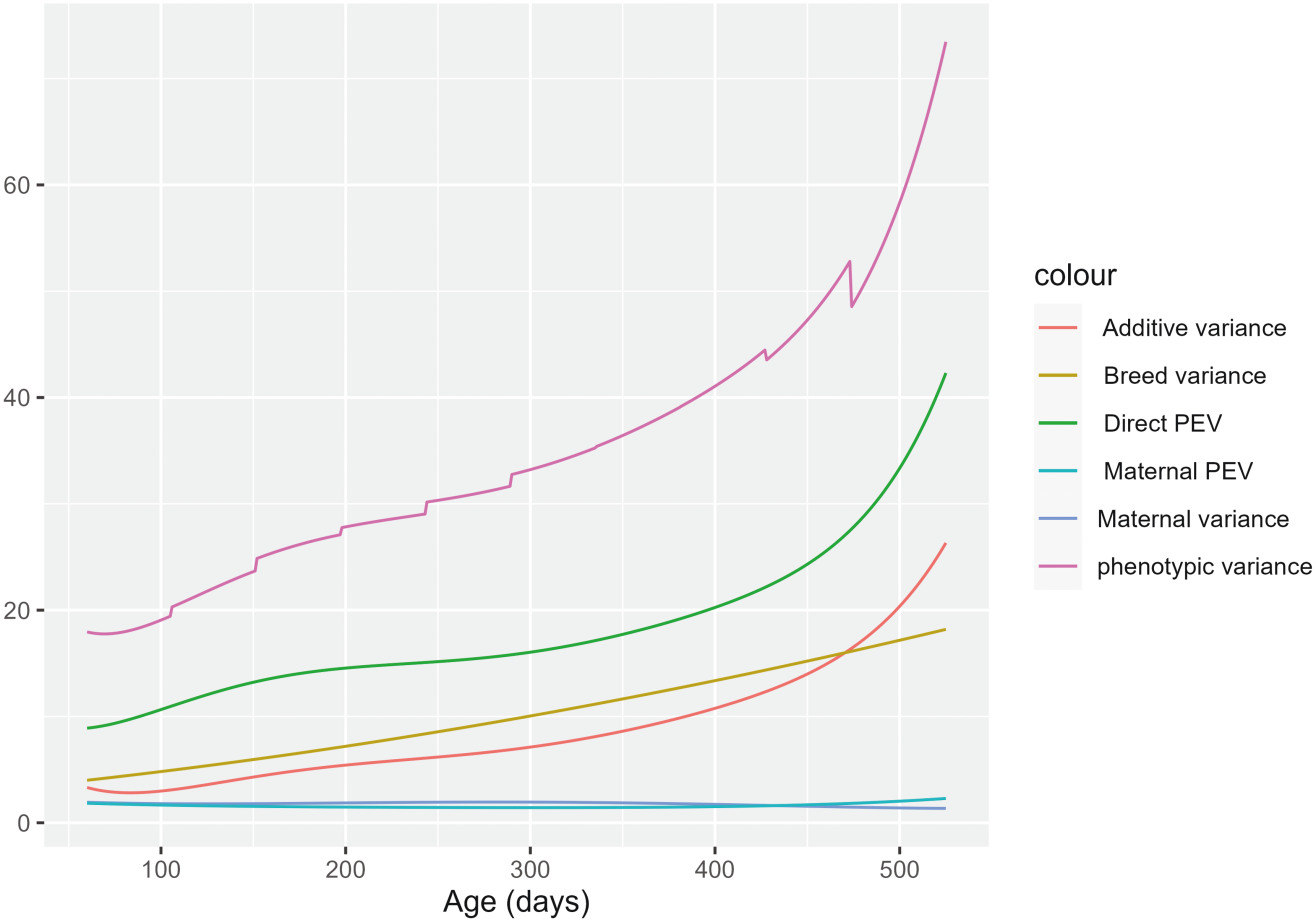
Variance parameters obtained from an RR model.

**Figure 3.**
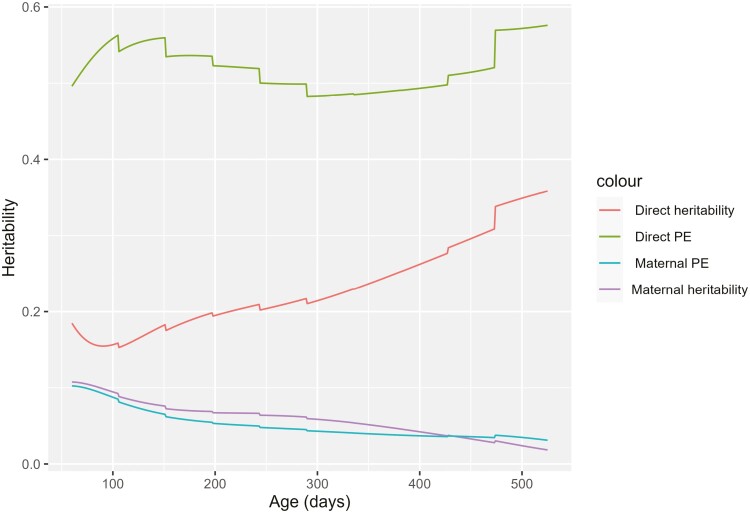
Heritability parameters obtained from an RR model.

Variance parameters and heritability estimates from the RR model are presented in Figures 2 and 3, respectively. Phenotypic variance increased steadily from 60 to 525 d. As phenotypic variance increased, additive genetic variance, animal permanent environmental variance, and breed variance also increased smoothly from beginning to end of the trajectory. By contrast, maternal genetic and maternal permanent environmental variance remains reasonably constant from the start to the end of the trajectory. Direct heritability also increased from the beginning to the end of the trajectory and was in the range of 0.15 to 0.35. By contrast, the maternal genetic and maternal permanent environmental effect ratio relative to the phenotypic variance decreased from the start to the end of the trajectory as expected (Figure 3).

### Variance parameters from the RR and the MT model

Estimates of variance parameters for fixed age points from both RR and MT models are presented in Tables 4 and 5, respectively. The phenotypic variance of weight increased from Wwt to Hwt from both methods. Direct heritability was lowest for Wwt (0.15) and highest for Hwt (0.28) from the MT model. Slightly higher estimates of heritability were observed from an RR model but were nonsignificant for Wwt and Ywt (*P* < 0.05). The variance of maternal effect decreased slightly with the use of the RR model.

**Table 4.** Variance parameters estimates from a MT model

Traits	*V*_a_	*V*_m_	*V*_mp_	*V*_p_	*V*_gg_	*h* ^2^
Wwt	3.02	1.93	1.86	19.63	4.68	0.15 ± 0.02
Pwt	4.84	2.33	1.87	27.28	6.12	0.17 ± 0.02
Ywt	9.19	1.98	1.48	38.34	12.06	0.24 ± 0.02
Hwt	14.35	1.72	2.23	49.97	17.16	0.28 ± 0.03

**Table 5.** Variance components estimates from the RR model

Traits, days	*V*_a_	*V*_pe_	*V*_m_	*V*_mp_	*V*_p_	*V*_gg_	*h* ^2^
100	2.98	10.65	1.80	1.67	19.06	4.81	0.16 ± 0.02
225	5.82	14.87	1.91	1.45	28.57	7.86	0.20 ± 0.02
360	8.99	18.15	1.85	1.45	37.22	11.98	0.24 ± 0.02
450	13.99	24.32	1.55	1.69	47.28	15.21	0.30 ± 0.02

### Genetic correlations

Genetic correlations between different weight stages are presented in Table 6. The genetic correlation decreased as the time between them increased; the genetic correlation between Wwts to other weight stages decreased from 0.83 to 0.64 from Pwt to Hwt from the MT model. A similar trend was also observed for the RR model. The genetic correlation between different weight stages was slightly different between the MT model and the RR model. Different genetic correlation values also indicate that variance and covariance observed from the RR model are somewhat different from that of the MT model.

**Table 6.** Genetic correlation between different weight stages; MT model above diagonal and RR model below diagonal

Age, days	Wwt	Pwt	Ywt	Hwt
100		0.83	0.64	0.64
225	0.82		0.74	0.70
360	0.72	0.87		0.89
450	0.71	0.79	0.87	
525	0.63	0.73	0.79	0.93

### Correlation of EBVs

**Figure 4.**
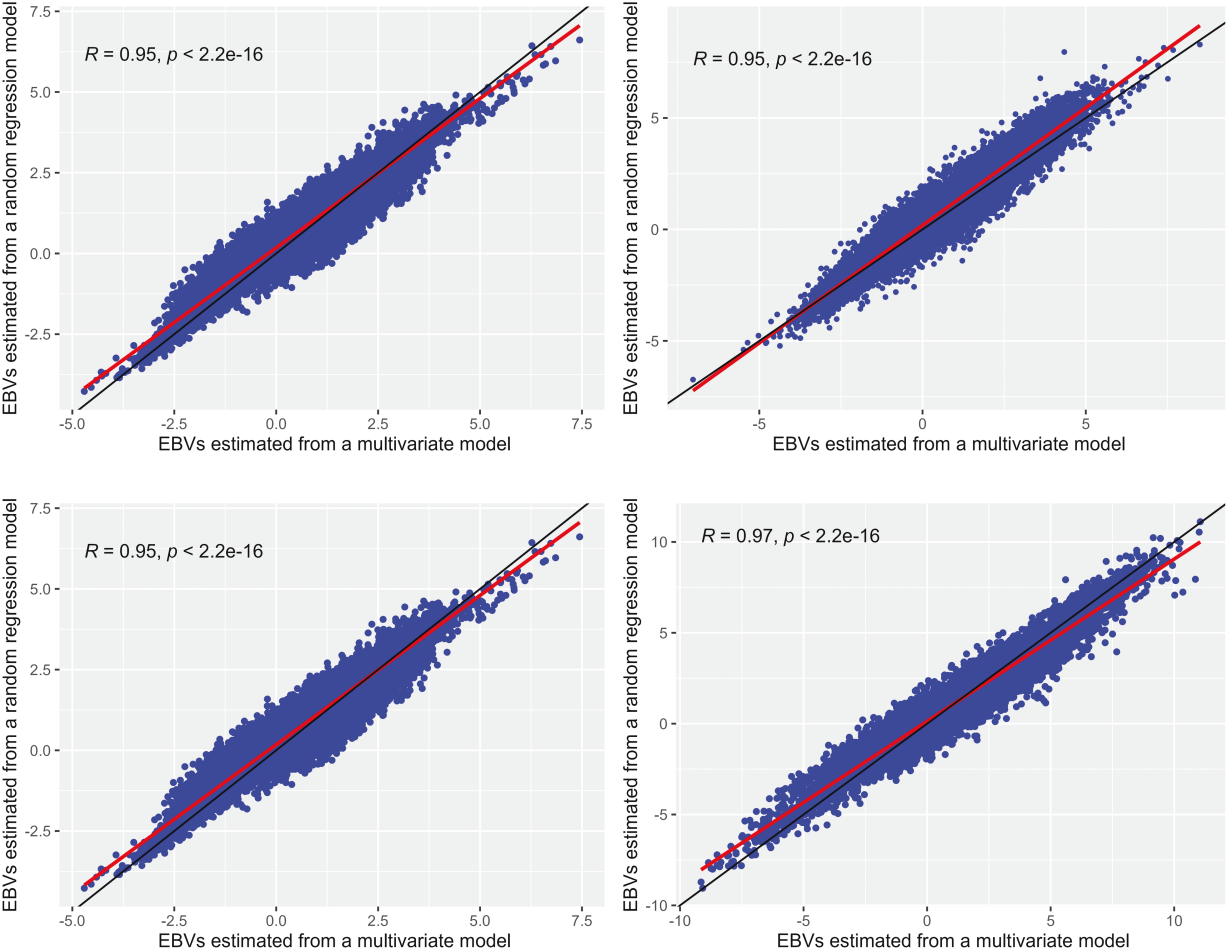
Correlation of EBVs estimated from an MT model to those estimated from an RR model (from top left to bottom right; Wwt, Pwt, Ywt, and Hwt).

The correlation of EBVs estimated from the MT model and those estimated from the RR model are presented in Figure 4 for each weight stage. Correlations of EBVs were presented because we are interested to know whether the re-ranking of EBVs occurs between the two methods. Correlations of EBVs were very strong, 0.95, 0.95, 0.95, and 0.97 for Wwt, Pwt, Ywt, and Hwt stages, respectively, indicating that the re-ranking of animals was less affected when EBVs are estimated from one method over another. However, the correlation of EBVs was significantly different from unity (*P* < 0.001), indicating that there could be a difference in the predictive ability of one method over another.

### Empirical validation of EBVs

Accuracy and slope estimate of EBVs for both MT and RR models are presented in Table 7. The accuracy and slope estimates observed in the RR model were not significantly different (*P* > 0.05) from those observed in the MT model.

**Table 7.** Accuracy and slope estimate of EBVs through forward prediction

Traits	MT model		RR model	
	Accuracy	Slope	Accuracy	Slope
Wwt	0.58 ± 0.02	0.91 ± 0.06	0.56 ± 0.02	0.91 ± 0.06
Pwt	0.51 ± 0.02	0.85 ± 0.06	0.51 ± 0.02	0.80 ± 0.06
Ywt	0.54 ± 0.02	0.80 ± 0.05	0.54 ± 0.02	0.79 ± 0.05
Hwt	0.56 ± 0.02	0.84 ± 0.07	0.54 ± 0.02	0.96 ± 0.08

Accuracy estimates of EBVs were also calculated from the standard error of prediction (SEP) to examine which method produces a better theoretical accuracy of EBVs (Table 8). We are further interested in how theoretical accuracy aligns with the empirical accuracy obtained from the validation method. Accuracy estimates obtained from the RR model were 0.70, 0.73, 0.81, and 0.54 for Wwt, Pwt, Ywt, and Hwt and were significantly higher than an MT model except for Hwt.

**Table 8.** Accuracy estimate of EBVs derived from the SEP

Traits	MT model	RR model
	Accuracy	Accuracy
Wwt	0.49 ± 0.001	0.70 ± 0.001
Pwt	0.52 ± 0.001	0.73 ± 0.001
Ywt	0.57 ± 0.001	0.81 ± 0.001
Hwt	0.57 ± 0.001	0.54 ± 0.001

### Running time

RR took slightly more time for the analysis, 7 h 50 min than the MT model, which took 5 h 40 min for variance component estimation for the given data. Similarly, RR took more time, 5 min than the MT model, 3 min to run the best linear unbiased prediction evaluation of breeding values.

## Discussion

Repeated weight records between 60 and 525 d of age from Australian meat sheep were analyzed to investigate how much improvement can be made using RR over the MT model and hence, whether the RR model should be used in the Australian sheep genetic evaluation. The RR model allowed variance and covariance of weight measurements to vary continuously across the growth trajectory. The RR model also resulted in an increase in additive genetic, breed, residual, and phenotypic variances with change in age. In contrast, maternal variance and maternal permanent variance remain nearly constant with age, which was also found in earlier studies (Fischer et al., 2004; Safari et al., 2005; Brown et al., 2016). The MT analysis produced point estimates of variance parameters at defined ages during Wwt, Pwt, Ywt, and Hwt stages. Heritability estimates obtained from the RR model are similar to those estimated from the MT model, with slightly higher estimates for the earlier ages. While the RR model allows variances and covariances to change gradually along the trajectory, it could not produce extra benefit in the predictive ability of breeding values. Extra benefits from the RR model can be achieved with additional information. In our data, most animals have records at all weight stages. The data set for the RR model had 15% more records than the MT model. More records might be needed to achieve a greater difference in the accuracy of EBVs. Automatic milking in dairy cattle with many records could benefit more from RR as some herds have daily records (Jamrozik et al., 1997; Van der Werf et al., 1998; Jensen, 2001; Guo et al., 2002; Strabel et al., 2005). If weight was measured more frequently in some flocks, the RR model could more easily accommodate this extra information and including multiple records from the same animals at each age stage could give it an advantage over the MT model, which uses only a single record for each weight stage.

The RR model took longer to compute than the MT model. Computation time can be reduced by either using a machine with higher processing power or using a lower-order RR model. Reducing the order of RR models can reduce the time without significantly changing the accuracy of prediction (Li et al., 2020; Paneru et al., 2022). Therefore, the RR model could be a suitable alternative to an MT model for weight traits, given the ram breeders’ increasing trend of multiple weight recording. The RR model has appealing properties of producing a smooth function of variance and covariance over a trajectory, making it less sensitive to measurements that are not around an average age in an age stage. Further, there could be bias for groups of animals at the edge of the weight stage in an MT model, and this would not be the case with the RR model.

### Variance parameters and correlations

Variance components observed from the current study using the MT model agree with the estimates from (Brown et al., 2016) in multi-breed meat sheep, except for Wwt, where they got slightly lower estimates (0.12 vs. 0.15 in our analysis). These parameters also agree with a weighted mean from seven and nine estimates for Wwt and Pwt from a literature review by (Safari et al., 2005).

Further, variance parameters obtained in this paper through the RR model are more consistent than estimates from Fischer et al. (2004), who observed a very high heritability at the end of the trajectory. This could be because of fewer observations at the end of the trajectory. Direct heritability was observed to be slightly higher at the beginning of the trajectory than around 100 d because of fewer records and higher-order polynomials. This emphasizes the need for sufficient data at the beginning and end of the trajectory. Similar behavior of the covariance function where the least data were present at the end of the trajectory was also demonstrated by Meyer (2001); Fischer et al. (2004). Meyer (2004) suggested that the “end range” problem observed in the covariance function can be avoided if the majority of the animals had a minimum number of records equal to the order of polynomial fit for direct and permanent environmental effects explored. The uniformity of the data all over the growth curve was observed as an important factor.

Genetic correlation between different weight stages decreases as the time between them increases because the trait is similar when they are very close and is less related when they are further apart. The RR model assumes all weight records as the same but correlated trait, taken at different time points. Genetic correlation decreased as the time between measurements increased, and a smooth trajectory was observed from the RR model. Genetic correlations observed in this study from the MT model were similar to previous literature estimates in sheep (Safari et al., 2005; Brown and Swan, 2016). A benefit of using an MT model is the ability to estimate correlations between weight traits and other performance indicators (such as carcass traits). This benefit will be greater if the different traits are favorably correlated. This is not directly feasible with the RR model.

### Correlation of EBVs

Genetic correlations between EBVs estimated from the RR model and those estimated from the MT model were very high for all weight stages. A high correlation of EBVs suggests that there will be less re-ranking of animals when EBVs are estimated from one method over another. Fischer et al. (2004) observed a lower correlation of EBVs: 0.82 and 0.87 for Wwt and Pwt in Australian meat sheep because EBVs were estimated from the univariate analysis. This study better reflects the current sheep genetic evaluation because EBVs were estimated from the MT model in the current evaluation (Brown et al., 2018; Collison et al., 2018).

### Validation of model-derived EBVs

Accuracy of the EBVs was also estimated through the SEP from the RR as well as the MT model. These estimates depend on the correctness of the genetic parameters used in the model. The RR model produced EBVs substantially more accurately than an MT method. The accuracy of EBVs was poorly estimated for Hwt using the RR model because the error variance decreased sharply at the end of the trajectory. This causes PEV to increase sharply, which might be a limitation of the RR model when fitted with a higher-order polynomial. However, accuracy estimated from SEP is affected by estimated parameters from the model used. Many papers on comparison between the RR and the MT model used SEP as a method of comparison (Boligon et al., 2011; Meyer, 2004). Inference made from that method may be biased as it depends on the genetic parameters used (and generated) by the model. We proposed to compare the accuracy of methods empirically by forward prediction.

Validation of EBVs is affected by heritability, selection, genetic groups in the model, and how animals in the validation are corrected for the fixed effects (Legarra and Reverter, 2017). It was expected that the RR model could not perform better because the phenotypes of the validation animals were corrected for fixed effects with solutions from a mixed model. An alternative is to estimate EBVs from the partial and full set as before, and the forward prediction was made using the method (Legarra and Reverter, 2017, 2018). In this method, accuracy is estimated by correlating EBVs estimated from the partial set to EBVs estimated from the complete set (true EBVs) for animals in the validation. Further, EBVs estimated from the partial set for the animals in the validation were regressed on EBVs estimated from the complete set (true EBVs) to estimate the slope. Even with this method, RR and MT models produced very similar accuracies, which illustrates that the choice of the method based on predictive ability is less influenced by how phenotypes are corrected for the fixed effects.

## Conclusion

The RR is more flexible to accommodate multiple weight records along the growth trajectory and from a theoretical point of view is more accurate in defining the covariances between these measurements, compared with the MT model. However, empirical validation, as well as correlations between breeding values estimated via RR and MT models, were high and both methods showed similar predictive ability for body weight measurements of sheep. The biggest advantage of RR over the MT model may be the ability to incorporate multiple records at any point in the trajectory. The RR model would be superior over the MT model if a large number of measurements can be recorded per animal with automated measuring devices.

## Abbreviations

AIC: Akaike’s information criterion
BIC: Bayesian information criteria
CG: contemporary group
EBVs: estimated breeding values
Hwt: hogget weight
LAMBPLAN: Australian national genetic evaluation for meat sheep
LRT: log-likelihood ratio test
MT: multiple trait
PEV: prediction error variance
Pwt: postweaning weight
RR: Random regression
SEP: standard error of prediction
Wwt: weaning weight
Ywt: yearling weight
